# Incidental Finding of Malpositioned Pacing Lead in the Left Ventricle in a Patient With Subacute Subdural Hematoma

**DOI:** 10.4021/cr192w

**Published:** 2012-07-20

**Authors:** Asma Syed, Sohail Salim, Ricardo Castillo

**Affiliations:** a Brookdale Hospital Medical Center, Brooklyn, NY, USA; b SUNY Downstate Medical Center, Brooklyn, NY, USA; c 1 Brookdale Plaza, Brooklyn, NY 11212, USA

**Keywords:** Pacemaker, Anticoagulation, Ventricular lead

## Abstract

Malposition of the right ventricular lead into the left ventricle is an unusual complication of challenging management. We report a case of an elderly woman with a dual chamber permanent pacemaker implanted 2 months before admission because of high grade AV block, who presented to our institution with sub acute subdural hematoma along the left fronto-parietal area. Incidental ventricular pacemaker lead in the left ventricle was found on chest CT scan. The patient was not candidate for anticoagulation due to her recent subdural hematoma, hence a discussion about the risks of explantation of the pacemaker lead led to patient’s lead extraction without any complication.

## Introduction

We are seen an increasing number of pacemaker implants nowadays, this is believed to be secondary to an increase in the number of elderly population and newer indications [1]. Pacemaker implantation is a procedure done routinely with few possible complications. Malposition of the right ventricular lead (RVL) is a known complication. However, malposition of the RVL into the left ventricle is an unusual complication [2, 3]. We report a case of a patient with malposition ventricular pacemaker lead presented with associated subdural hematoma.

## Case Report

An 89-year-old woman with history of hypertension and high grade AV block, for which she underwent a dual chamber permanent pacemaker insertion 2 months before; presented to our institution with complaints of slurred speech along with left sided body weakness of 1 day duration. The patient did not give history of fall preceding the above complaints.

**Figure 1:**
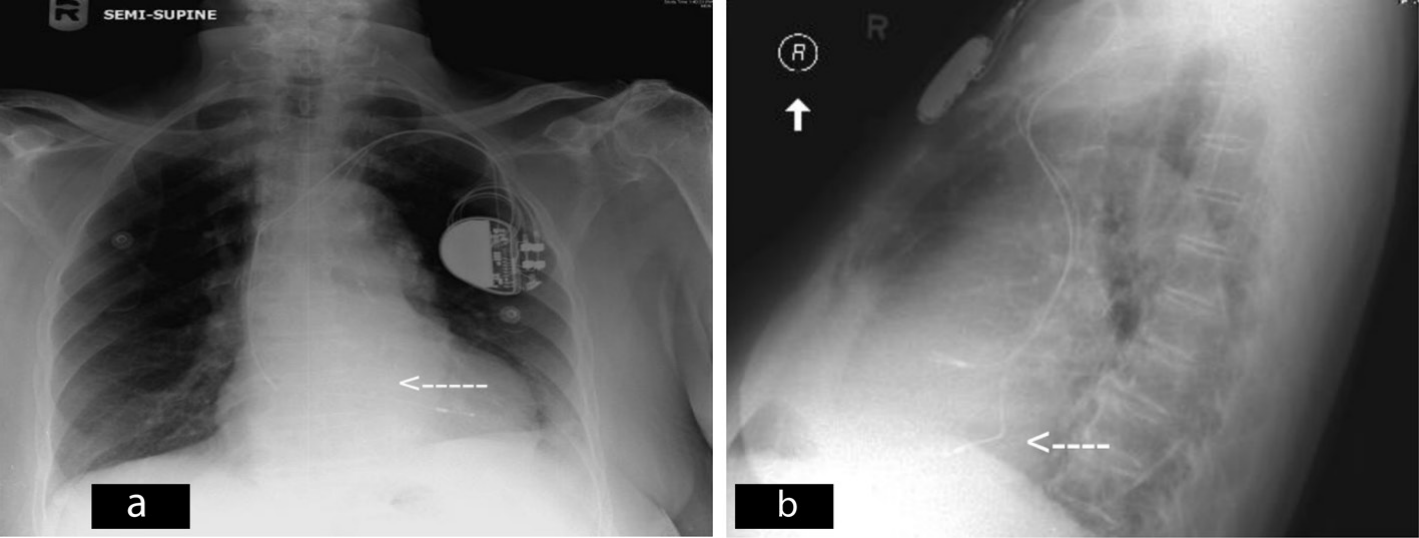
a: chest X ray demonstrating the ventricular lead crossing the spine at a higher level (white arrow); b: lateral Chest X ray, revealing suspicious ventricular lead position (white arrow).

Admission head CT scan showed a sub acute subdural hematoma along the left fronto-parietal convexity, chest-x-ray revealed a dual chamber pacemaker with the ventricular lead noted to cross the spine higher than expected (Fig 1a, b) and the electrocardiogram (EKG) showing right bundle branch block pattern (RBBB). Incidental finding of pacemaker ventricular lead malposition then was suspected.

**Figure 2:**
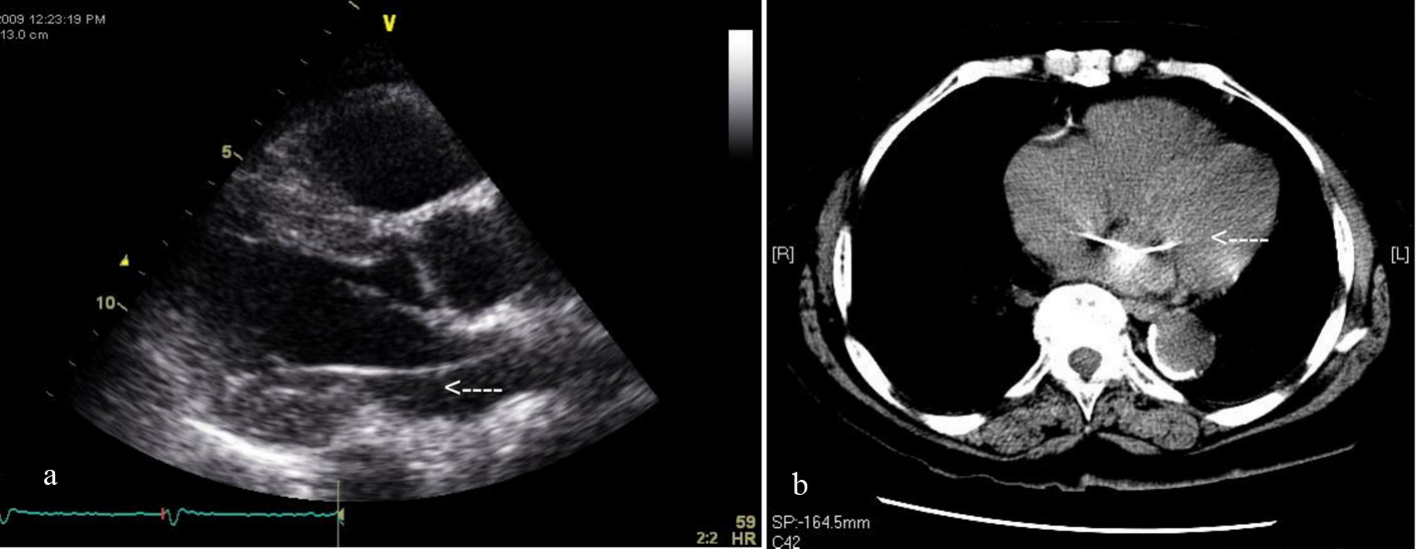
a: echocardiogram showing the presence of the ventricular pacing lead in the left ventricle; b: CT scan of the chest demonstrating the ventricular lead crossing the inter-atrial septum.

Transthoracic echocardiogram showed the ventricular pacemaker lead crossing the inter-atrial septum into left atrium and down into the left ventricle, there was no evidence of thrombus and the left ventricular systolic function was normal(Fig 2a). A chest CT scan confirmed the malpositioned ventricular pacemaker lead (Fig 2b) and pacemaker interrogation showed appropriate device functioning.

The patient’s symptoms progressively improved and she was managed conservatively without hematoma evacuation. Although, the ventricular pacemaker lead could have been left undisturbed, the patient was not candidate for anticoagulation due to her recent subdural hematoma, hence a discussion about the risks of explantation of the pacemaker lead led to patient’s lead extraction without any complication.

## Discussion

Permanent pacemaker implant is a common and increased in demand procedure. In 2002, 612 new implants per million of habitants were performed in the United States, almost twice the number performed 12 years before [1]. Despite this fact there still exists an infrequent list of complications associated with it, including lead malposition [4, 5]. Review of the literature shows that a malpositioned pacemaker lead accounts for as high as 6% of the complications. In our case it was unclear whether the patient had a preexisting foramen ovale or the pacemaker lead itself perforated the interatrial septum. Most reported cases show that common routes of malpositioned leads are through the interatrial septum but other routes such as perforated ventricular septum or retrograde implantation via subclavian artery have also been reported [4]. If EKG shows paced RBBB, there should be a high index of suspicion of malpositioned lead and thorough investigation should be performed. Chest x ray alone could be unclear but chest CT scan or echocardiography would determine lead position. Review of literature has showed that there is an increased risk of thrombus formation on the lead and patients should be fully anticoagulated, but there are no guidelines as there is limited data available. In case where the patient may not be able to tolerate full anticoagulation, pacemaker lead revision should be considered. Benefits versus risks should be weighed and a case specific approach should be implemented.
